# Commentary: Vasopressin-induced hyponatremia in infants <3 months of age in the neonatal intensive care unit

**DOI:** 10.3389/fped.2024.1514079

**Published:** 2025-01-20

**Authors:** A. Hebert, D. R. Rios, P. J. McNamara

**Affiliations:** ^1^ Division of Neonatology, CHU de Québec, Université Laval, Quebec City, QC, Canada; ^2^ Division of Neonatology, University of Iowa Children’s Hospital, University of Iowa, Iowa, IA, United States

**Keywords:** vasopressin, neonates, hypotension, hemodynamics, hyponatremia

A Commentary on Vasopressin induced hyponatremia in infants <3 months of age in the neonatal intensive care unit By Patel K, Thomson S, Vijayan M, Makoni M, Johnson PN, Stephens K, Neely SB and Miller JL (2024). Front. Pediatr. 12:1465785. doi: 10.3389/fped.2024.1465785

We read with great interest the article by Patel et al. on “Vasopressin-induced hyponatremia in infants <3 months of age in the neonatal intensive care unit” published in Frontiers in Pediatrics (1). The authors have contributed valuable insights into the potential side effects, such as hyponatremia, of vasopressin use in neonates. Their findings underscore the importance of careful monitoring when vasopressin is used in neonatal intensive care units. On the contrary, clinicians should be cognizant of balancing these unintended risks and their impact on neonatal outcomes against the overall beneficial and potentially life-saving effects on pulmonary hemodynamics and right heart function.

Of note, while the risk of hyponatremia is an important consideration, the end-organ consequences of vasopressin-related hyponatremia remain unknown. It is well recognized, however, that acute and severe hyponatremia may lead to serious complications, including cerebral edema, seizures, worsening of respiratory distress, and potential long-term neurodevelopmental impairments (2). To better understand the clinical relevance of the observed hyponatremia we pose the following questions. *First*, are there data to suggest that hyponatremia, as described in this cohort, contributed to any adverse clinical outcomes in the index population? *Second*, did the presence of hyponatremia necessitate adjustments in clinical care, and if so, how were these addressed by the clinical team? Understanding whether interventions were employed to mitigate hyponatremia and whether these interventions led to improved outcomes would add depth to the discussion of the risks and benefits of vasopressin therapy. Prior studies on the use of vasopressin in newborns with refractory acute pulmonary hypertension provide some insights. For example, Ouellet et al. (3), reported cases of hyponatremia but without evidence of potentially attributable clinical complications, such as seizures, worsening of intraventricular hemorrhage, and respiratory distress.

It is noteworthy that, although not statistically significant, the incidence of hyponatremia was lowest in the last year of the study period. Readers may wonder whether this decrease is related to the learning curve related to vasopressin use, changes in the duration of vasopressin use over time, or adjustments in dosing protocols. Understanding the biologic nature of hyponatremia is an important consideration. At first glance, the nature of hyponatremia may be presumed to relate to decreased urinary output; however, higher doses of vasopressin promote natriuresis (4). It would have been helpful to explore the relationship between the incidence of hyponatremia and maximal and cumulative dose of vasopressin, sodium intake, and urinary sodium losses. Therefore, it would be of interest to know if corrective measures for hyponatremia, such as fluid restriction or sodium supplementation, were systematically employed. Capolupo et al. (5) described how early vasopressin infusion improved oxygenation in infants with congenital diaphragmatic hernia. In their cohort, resulting hyponatremia was managed in all 18/27 infants (66.7%) with fluid restriction: of them, 16/18 (88.9%) also received a careful supplementation of serum sodium during vasopressin administration (5). Hyponatremia in this context can result from either salt loss, due to renal sodium wasting, or water retention, often related to vasopressin's antidiuretic effects (2). Understanding the underlying mechanism is crucial, as management strategies differ; for example, water retention-induced hyponatremia may be managed with fluid restriction, while early and aggressive sodium supplementation is indicated in cases of salt loss. It is plausible that modification to care practices over time, either on an individual care basis or thought introduction of standardized care guidelines, may have impacted the frequency of hyponatremia.

In summary, clinicians should be cognizant of the balance between managing side effects and optimizing the desired therapeutic effect which is key in critical care management, particularly in the NICU setting. Vasopressin remains a critical therapeutic agent in neonates, particularly for managing refractory hypotension and pulmonary hypertension; however, evidence remains limited to observational studies and clinical trials are lacking. Nevertheless, the potential benefits of vasopressin may outweigh the risks in some patients; therefore, accepting the possibility of hyponatremia and implementing strategies to mitigate risk may be a necessary sacrifice to achieve cardiovascular stability. We commend the authors for their important contribution to understanding the side effects of vasopressin in neonates, and we hope that these additional considerations can further enrich the discussion surrounding the use of Vasopressin in this population.
