# Erratum: Commentary: Vasopressin-induced hyponatremia in infants <3 months of age in the neonatal intensive care unit

**DOI:** 10.3389/fped.2025.1593148

**Published:** 2025-03-24

**Authors:** Frontiers Production Office

**Affiliations:** Frontiers Media SA, Lausanne, Switzerland

**Keywords:** vasopressin, neonates, hypotension, hemodynamics, hyponatremia

An erratum on Commentary: Vasopressin-induced hyponatremia in infants <3 months of age in the neonatal intensive care unit By Hebert A, Rios DR and McNamara PJ (2025). Front. Pediatr. 12:1514079. doi: 10.3389/fped.2024.1514079

Due to a production error, this article was published with the incorrect title.

The correct title appears below:

“Commentary: Vasopressin-induced hyponatremia in infants <3 months of age in the neonatal intensive care unit”

The publisher apologizes for this mistake.

The original version of this article has been updated.

